# Chitosan-Based Nanoparticles for Nucleic Acid Delivery: Technological Aspects, Applications, and Future Perspectives

**DOI:** 10.3390/pharmaceutics15071849

**Published:** 2023-06-29

**Authors:** Maria Karayianni, Theodore Sentoukas, Athanasios Skandalis, Natassa Pippa, Stergios Pispas

**Affiliations:** 1 Theoretical and Physical Chemistry Institute, National Hellenic Research Foundation, 48 Vassileos Constantinou Ave., 11635 Athens, Greece; mkaragia@eie.gr (M.K.); thanos.skan@gmail.com (A.S.); 2 Centre of Polymer and Carbon Materials, Polish Academy of Sciences, 34, M. Curie-Sklodowska St., 41-819 Zabrze, Poland; tsentoukas@cmpw-pan.pl; 3 Section of Pharmaceutical Technology, Faculty of Pharmacy, Panepistimioupolis Zografou, National and Kapodistrian University of Athens, 15771 Athens, Greece; natpippa@pharm.uoa.gr

**Keywords:** chitosan, nanoparticles, DNA, RNA, nucleic acid delivery, gene therapy, polyelectrolyte complexation, polyplex

## Abstract

Chitosan is a naturally occurring polymer derived from the deacetylation of chitin, which is an abundant carbohydrate found mainly in the shells of various marine and terrestrial (micro)organisms. Chitosan has been extensively used to construct nanoparticles (NPs), which are biocompatible, biodegradable, non-toxic, easy to prepare, and can function as effective drug delivery systems. Moreover, chitosan NPs have been employed in gene and vaccine delivery, as well as advanced cancer therapy, and they can also serve as new therapeutic tools against viral infections. In this review, we summarize the most recent developments in the field of chitosan-based NPs intended as nucleic acid delivery vehicles and gene therapy vectors. Special attention is given to the technological aspects of chitosan complexes for nucleic acid delivery.

## 1. Introduction

Recent developments in the field of biomedical nanotechnology offer new pathways against infectious diseases that are the leading cause of human morbidity and mortality. Infections caused by bacteria, viruses, fungi, and parasites are responsible for a vast number of deaths annually worldwide [1]. They remain one of the main health concerns and threats globally with substantial social and economic impacts. The successful prevention and treatment of various types of infections is one of the primary concerns of modern medicine. For this reason, the development of novel methods and materials suitable for biotechnological applications aiming at different therapeutic routes is always essential. One particular category of materials that has attracted broad interest in this regard is that of nanoparticles (NPs) of either organic or inorganic origin. With sizes below 100 nm, they have special properties due to their high surface-area-to-volume ratio and have been proven to be very advantageous when it comes to nanomedicine and biomedical applications such as drug and gene delivery vectors, antimicrobial agents, tissue engineering, wound healing scaffolds, anticancer nanosystems, and therapeutic delivery platforms [1,2,3]. NPs are produced using either top-down procedures, such as sonication, high pressure, and homogenization, or bottom-up processes, such as solvent displacement and reactive precipitation [2,4]. Furthermore, polymeric NPs can be constructed from natural and synthetic polymers, offering additional advantages since they are characterized by increased stability and ease of surface modification [2,4]. Biopolymer NPs which intrinsically exhibit biocompatibility, biodegradability, and reduced toxicity hold especially great promise for bioapplications.

**Figure 1:**
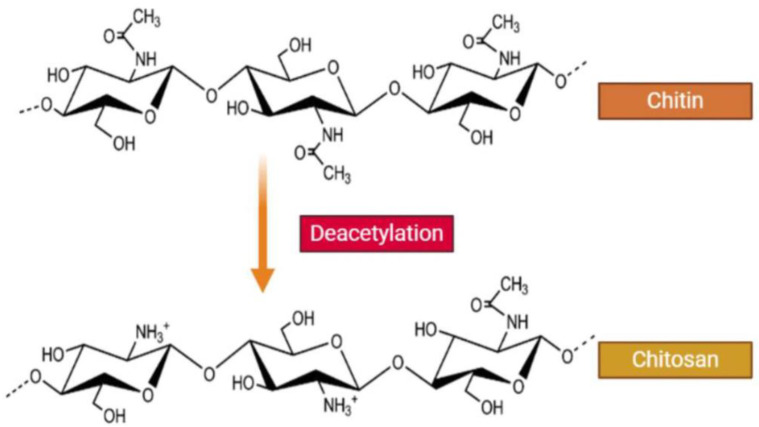
Chemical structure of chitin and chitosan (adapted from [1]).

The ever-growing need for natural polymers that can serve as building blocks for developing nanomaterials relevant to various biological applications has attracted particular interest in naturally occurring carbohydrates and polysaccharides. One such example is chitosan, which is derived from the deacetylation of chitin (Figure 1), the second most abundant biopolymer in nature since it represents the primary component of marine crustaceans (i.e., shrimp, crab, lobster), cuticles of insects, and cell walls of micro-organisms [3,5]. Chitosan has a cellulose-like carbohydrate structure composed of d-glucosamine and *N*-acetyl glucosamine alternating repeating units, linked by a *β*-(1→4)-glycosidic linkage [2,4,6,7]. Directly correlated to the biocompatible, biodegradable, and non-toxic structure of chitosan are its unique antimicrobial and mucoadhesive properties that render it ideal for a plethora of biotechnological functions. Apart from its uses in the biomedical industry and genetic engineering, chitosan has also been proven valuable in agriculture, the food manufacturing sector, environmental pollution control, water treatment, paper manufacturing, cosmetic products, and photography, among others [2,4,6,8]. As one might expect, chitosan NPs have even greater applicability and utilization potential as they combine the intrinsic biorelevant properties of chitosan with the versatility and benefits of polymeric nanosized particles. They are relatively easily produced without the need for toxic organic solvent due to chitosan’s solubility in aqueous acidic solutions [9], while at the same time, they demonstrate interesting interface and surface effects, owing to their high surface-to-volume fraction [1,2]. Over the years, the use of chitosan NPs in numerous processes involving varying delivery approaches for drugs, genes, vaccines, and chemotherapeutic agents has been established [9].

Among the vast variety of possible biomedical applications of chitosan NPs, their employment as non-viral gene delivery vectors has lately gained increasing scientific momentum, ever since gene therapy emerged as a promising therapeutic approach for challenging genetic diseases [10,11,12]. One of the main objective difficulties of this approach is the successful delivery of the genes, owing to the fact that naked nucleic acids cannot cross the cell membrane, are easily degraded by serum nucleases, and bear non-specificity toward targeted cells [10,11]. Therefore, it is of predominant importance to develop safe and efficient gene delivery systems, and chitosan seems an excellent candidate for this task due to its cationic nature that enables electrostatic complexation with oppositely charged DNA and RNA chains. This subfield of biotechnological research is the main focus of this review, with the aim of highlighting the most current and significant scientific advances in utilizing chitosan and its NPs as nanocarriers for gene delivery systems.

## 2. Properties and Applications of Chitosan

Chitosan is obtained from the deacetylation of chitin by chemical or enzymatic hydrolysis, resulting in a linear cationic polysaccharide that exhibits reduced crystallinity and increased solubility compared to its chitin precursor [1,2,6,8]. The degree of deacetylation (DD) along with the molecular weight (*M*w) of the resulting chitosan polymer mainly determine its physicochemical and biological properties since they define the total number of primary amine residues [1]. Due to the presence of the protonatable amino groups, chitosan can be dissolved in dilute acidic (e.g., acetic, formic, hydrochloric, lactic, citric) solutions [6,7]. Moreover, chitosan contains numerous primary and secondary hydroxyl groups that further enhance interactions with water. In practice, the solubility is affected not only by the deacetylation degree and molecular weight of the chitosan chain (with higher DD and lower *M*w being more favorable) but also by the concentration, pH, and ionic strength of the solution, while agitation can assist in the dissolution process [1]. In addition to its biodegradability, biocompatibility, bioactivity, and non-toxicity, another important advantage of chitosan is that it can be processed in different forms, such as solutions, blends, sponges, membranes, gels, pastes, tablets, microspheres, or microgranules, depending on the intended application [1]. Chitosan’s solubility and overall properties can be additionally improved through appropriate either physical or chemical modification that is facilitated by the presence of the amino and hydroxyl active groups [5,6,7,8]. Physical modification usually entails mixing or blending with other hydrophilic polymers such as polyvinyl alcohol (PVA), polyethylene oxide (PEO), and polyvinyl pyrrolidone (PVP), thus generating a new polymeric material with different and distinct physical properties [8]. When it comes to chemical modification, the functional groups in the chemical structure of chitosan enable a plethora of reactions [7,8]. More specifically, etherification, esterification, cross-linking, graft copolymerization, and *O*-acetylation are carried out on the hydroxyl groups, while acetylation, quaternization, Schiff’s base reaction, and grafting are carried out on the amino groups [7]. The produced functionalized analogues are characterized by quicker gel-formation properties, greater aqueous solubility, and the capability of forming self-assembling nanostructures [6]. Consequently, chitosan derivatives have attracted considerable interest due to their tunable biological and chemical characteristics, along with their compelling application capabilities [13,14].

The chemical structure of the chitosan polymer greatly influences its overall physical properties as well, since each free amino group of the d-glucosamine monomer can also become positively charged, promoting its solubility and antibacterial activity [6]. Moreover, these moieties can serve as excellent chelating ligands and can potentially bind several metal ions, either through interaction with the free amino groups (at near-neutral pH) or by electrostatic attraction on protonated amino groups (in acidic solutions) [6,8]. Along the same lines, other ways of improving chitosan’s solubility entail lowering its *M*w and crystallinity [6]. The cationic nature of chitosan is also responsible for most of its corresponding biological properties and functionalities. For example, the antibacterial effect of chitosan is attributed to the interaction of its positively charged amino groups with the negatively charged phosphoryl groups of phospholipids at the bacterial cell wall, which changes their metabolism and eventually leads to cell death [7]. Moreover, it was established in various in vitro experiments that this antibacterial function could result from chitosan’s DNA binding capacity, in the sense that when it comes in contact with the nuclei of bacteria, it combines with DNA and prevents mRNA synthesis [8]. The same cytocidal mechanism is also responsible for the anti-fungal property of chitosan, which is influenced to a significant degree by its DD and *M*w (i.e., it is enhanced when the DD is higher and the *M*w is smaller) [8]. Another prominent biological property of chitosan is that it does not cause severe inflammation or stimulation of the immune system, or in other words it has low toxicity, as it has been established in several animal models [4,6,15,16,17]. Furthermore, it shows anti-inflammatory action by reducing the release of interleukin-8 (IL-8) and tumor necrosis factor (TNF) from mast cells and even an analgesic effect by reducing the concentration of inflammatory mediators (bradykinin) at the site of injury [8]. Chitosan also exhibits wound healing and hemostatic properties and stimulates the formation of granulation tissue and the activity of fibroblast proliferation, while at the same time it suppresses fibrosis and promotes erythrocyte adhesion, fibrinogen adsorption, and platelet adhesion and activation [7,8]. Ultimately, its innate biodegradability and biocompatibility are especially important properties since in biological organisms, bioenzymes can catalyze the depolymerization of chitosan with the breakdown products *N*-acetyl glucose and glucosamine, as well as the degradation intermediates, being non-harmful to the human organism [4]. Specifically in the human body, chitosan is known to be degraded mainly by lysozyme, a proteomic enzyme present in all human tissues, as well as lipase, which can be found in the human gastric or pancreatic fluid or saliva [18,19]. Moreover, several human chitinases, glucosidases, and proteases have been identified to have enzymatic activity and be able to degrade chitosan to varying degrees [20]. Nevertheless, the DD and *M*w of the biopolymer play a crucial role when it comes to its biodegradability, with the degradation of chitosan chains with lower DD and *M*w being more feasible [20,21].

On top of all the above, chitosan’s ability to form NPs with relative ease has been vastly exploited as a means of producing nanocarriers with exceptional characteristics that exhibit greater affinity for negatively charged biological membranes and thus can be used to effectively load drugs, enzymes, and nucleic acids [4]. In fact, various anticancer drugs, antimicrobials, peptides, anti-inflammatories, growth factors, and other pharmaceuticals have been successfully delivered using chitosan-based polymeric drug carriers [4]. Several methods are being utilized for the production of chitosan NPs, including ionotropic gelation, microemulsion, emulsification solvent diffusion, polyelectrolyte complex formation, and the reverse micellar method [1,2,4,9]. Among these methods, the most widely used ones are ionotropic gelation and polyelectrolyte complexation, which are simple and do not apply high shear force or use organic solvents [2]. In more detail, ionotropic gelation is based on the electrostatic interaction between the amine groups of chitosan and the negatively charged groups of polyanions such as tripolyphosphate (TPP), while a stabilizer such as a poloxamer may be also used. The size and surface charge of the formed ionically/electrostatically cross-linked NPs can be finetuned by changing chitosan’s concentration, chitosan-to-polyion or stabilizer ratio, and/or ionic strength of the solution [1,2]. In a similar manner, the polyelectrolyte complex method includes the addition of an anionic polymeric macromolecule such as nucleic acid (DNA and RNA) or a protein solution to a cation-based polymeric chitosan dissolved in acetic acid solution, with the consequent charge neutralization being assisted by mechanical stirring at room temperature [9]. The advantages of this method are its simplicity under typical conditions and the spontaneous formation of loaded NPs.

It comes as no surprise that the unique set of properties of chitosan and its corresponding NPs render them suitable for a wide range of biologically relevant applications of major significance. To name but a few, chitosan finds usage in drug delivery, anti-tumor and anti-cancer therapy, nucleic acid delivery and gene therapy, protein delivery, tissue engineering and regeneration, wound dressing and healing, bioimaging, and as an antimicrobial agent and a vaccine adjuvant but also in other fields such as food industry, wastewater treatment, cosmetics industry, and agriculture [1,2,3,4,5,6,7,8,9]. Especially regarding nucleic acid delivery for the design of the optimal polymeric delivery system, several factors should be taken into consideration. These include the ability to form a stable complex/nanoparticle upon coupling with the nucleic acids, protect the cargo from nuclease degradation, target the desired cells, and promote cellular entry. The preparation protocol of these delivery systems usually entails the mixing of diluted chitosan and nucleic acid solutions followed by incubation, thus generating chitosan/DNA or RNA complexes driven by strong electrostatic interactions, also known as polyplexes, that protect condensed nucleic acids from enzymatic degradation [10]. The properties of such polyplexes mostly depend on the structural properties and concentration of chitosan, the mixing ratio of the two components, and the pH value of the solution [10]. After entering the cell, the carrier and cargo must escape from endosomes and lysosomes, release the genes in the cytoplasm, and interact with target cellular elements while showing low toxicity [10]. By carefully selecting the properties of the chitosan biopolymer used for the formation of the DNA/RNA polyplexes and especially its *M*w, one can finetune the size and stability of the resulting complexes, along with the cellular uptake and release of genes inside the cytoplasm, and finally influence the transfection efficiency [10]. The use of chitosan as a gene delivery vehicle has been intensively studied due to its unambiguous advantages, for instance its cationic nature, biocompatibility, relatively low cost of production, and facile functional modification [10].

## 3. Technological Aspects of Chitosan Complexes for Nucleic Acid Delivery

The preparation of chitosan complexes for nucleic acid delivery and targeting is of paramount importance for the behavior of polyplexes in vitro and in vivo. Chitosan, with its highly positive charge, can easily interact with negatively charged nucleic acids, forming polymeric complexes—polyplexes [22,23,24,25,26]. There are three different ways of incorporating genetic material into chitosan and/or chitosan derivatives: encapsulation, adsorption, and electrostatic interactions. Each incorporation mechanism exhibits a different release profile and endosome escape route of the genetic material. Chitosan-based non-viral gene vectors can be fabricated in different morphologies and shapes, ranging from nanoparticles and nanocapsules to micelles, each of which exhibits unique physicochemical and biological properties, as well as loading and release properties.

Several parameters affect the technology and the properties of the nanoparticulate systems self-assembled from chitosan [22,23,24,25,26]. Firstly, the molecular weight of chitosan is one of the most crucial parameters for the design and development of chitosan gene vectors. The size of pure nanoparticles, the size of the complexes, the physicochemical stability, the transfection efficiency, and the targeting to the subcellular organelles can be altered by the different molecular weights of chitosan [27,28]. Secondly, the molar stoichiometry of the mixed chitosan/nucleic acid which is expressed as the ratio of chitosan nitrogen N per gene phosphate. This molar ratio is an important formulation attribute for the stability of the polyplex, its interaction with the cellular membrane by the endocytosis mechanism, and the transfection efficiency [27,28]. Thirdly, the pH of the dispersion medium, which is usually constant between 5.6 and 6.5. Alteration of the pH values leads to different surface characteristics of the chitosan nanoparticles and complexes, as well as their stability [22,23,24,25,26].

Various protocols for the preparation of chitosan nanoparticles are reported in the literature, i.e., ionic cross-linking, covalent cross-linking, reverse micellar method, etc. [10,29,30]. Brunel et al. [30] used a preparation protocol based on a reverse emulsion of a chitosan solution in a Miglyol/Span 80. The limitation of this method is that the degree of acetylation of chitosan should be lower than 30% and the advantage is the preparation of colloids with a “green” method controlled by the concentration of the surfactant and temperature. Emulsification has also been used as a preparation method that includes both the mechanical shaking and the high-pressure homogenization for the o/w emulsion [29]. Namely, the emulsification solvent diffusion method was used for the preparation of chitosan nanoparticles with the aid of lecithin and poloxamer 188 as emulsifiers. The prepared nanoparticles were around 100 nm with positive zeta potential. It should be noted that the reverse emulsion and the emulsification solvent diffusion method are used for the preparation of chitosan nanoparticles and not for the encapsulation of genetic material. For their loading with nucleic acids, further formulation steps are required [1].

On the other hand, two different fabrication protocols for the preparation of chitosan-based carriers for gene delivery are used widely in the literature [10]. The first one is based on the strong electrostatic interactions between the chitosan and the genetic material. This technique is also described as the polyelectrolyte complexation process. Soliman et al. [31] prepared nanoparticles composed of chitosan with different degrees of deacetylation and hyaluronic acids, incorporating mRNA via the polyelectrolyte complexation process. Namely, chitosan and hyaluronic acid stock solutions were dispersed to specific molar glucosamine 1 (N) to mRNA phosphate (P) to carboxyl (C) ratio (N:P:C) of 5:1:0, 5:1:1, and 5:1:7. The physicochemical characteristics of the complexes, as well as the transfection efficiency, were strongly dependent on the ratio of the components. The same preparation protocol was used by Nicolle et al. [32] for the development of covalent chitosan–polyethyleneimine derivatives as non-integrating DNA delivery systems.

**Figure 2:**
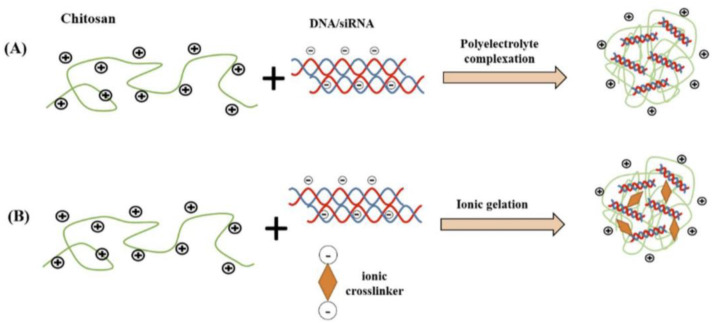
Common chitosan nanocarrier fabrication methods for nucleic acid delivery. (**A**) polyelectrolyte complexation; (**B**) ionic gelation (adapted from [10]).

The other method is the ionic gelation protocol, which needs an ionic crosslinker. For example, Cao et al. [33] designed an siRNA/chitosan–methacrylate complex with UV cross-linkable gels for prolonged gene silencing. Ionic gelation was used for the preparation of anti-rabies chitosan–DNA nanoparticles as vaccines [34]. Namely, chitosan compact particles were self-assembled by ionic gelation and conjugated by coacervation with a pDNA rabies vaccine to investigate their encapsulation and transfection efficiency. The resulting polyplexes exhibited a size close to 100 nm, positive zeta potential, and maximum attachment efficiency strongly dependent on the genetic material. In Figure 2, the two fabrication processes are visualized. In a recent study published in the literature, chitosan nanoparticles loaded with siRNA were prepared with the synergetic protocol, combining electrostatic complexation and chemical cross-linking for the treatment of melanoma in mice [35]. Namely, phenylboronic acid-modified chitosan oligosaccharide nanoparticles were used for the delivery of survivin-targeted siRNA. The size and the surface charge of the polyplexes were found to be dependent on the ratio of the components, but in all series of the formulation, the particulate systems were below 400 nm with zeta potential values. These nanosystems significantly inhibited the proliferation of cell lines in vitro and also inhibited the growth and metastasis of melanoma in mice.

The codelivery of transcription 3 siRNA and BV6, a well-known inhibitor of apoptosis, was achieved by the combination of the aforementioned methods for carboxymethyl dextran trimethyl chitosan nanoparticle preparation. In this case, the nanoparticles were around 100 nm, with low polydispersity and a positive surface charge [36].

**Figure 3:**
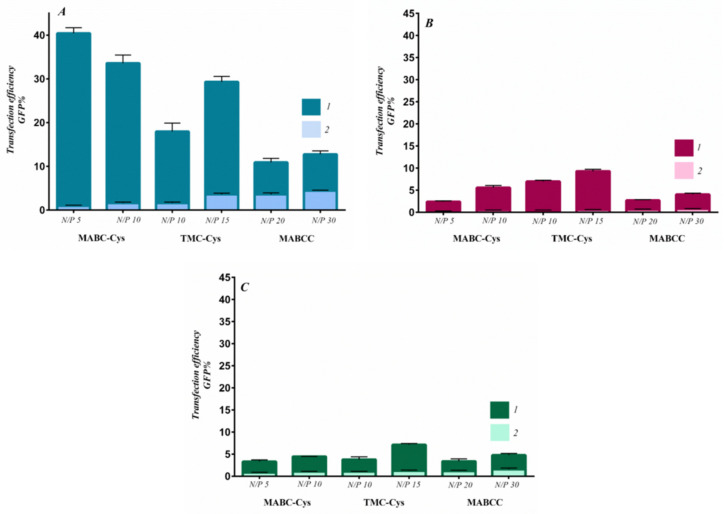
Transfection efficiency of MABC-Cys, TMC-Cys, and MABCC polymers represented as the percentage of cells expressing GFP using the starvation method (method 2) in (**A**) HEK-293T, (**B**) SKOV3, and (**C**) MCF-7 cell lines. 1: Chitosan derivative; 2: base chitosan. Data shown as mean ± SEM. (Thiolated trimethyl chitosan: TMC-Cys; methylated 4-*N*,*N* dimethyl aminobenzyl *N*,*O* carboxymethyl chitosan: MABCC; and thiolated trimethyl aminobenzyl chitosan: MABC-Cys.) (Adapted from [24]).

Different chitosan derivatives were synthesized and were complexed with plasmid DNA in different N/P ratios via the ionic gelation method. The size, size distribution, zeta potential, and transfection efficiencies were evaluated in terms of N/P ratios of each chitosan derivative. The outcomes showed that all the chitosan derivatives could encapsulate plasmid DNA at N/P equal to two. The majority of polyplexes exhibited a size of around 120 nm, a spherical shape, and a positive surface charge ranging from 10 to 30 mV. In Figure 3, the dependence of the N/P ratio on the transfection efficiency is presented for the different chitosan derivatives [24].

The design of this experiment (D-optimal design with two factors) has been also used in the literature for the development of chitosan-based polyelectrolyte nanoparticles for gene delivery. The independent variables were the concentration of trimethyl chitosan and the type of negatively charged polyelectrolyte, while the factors were the size, size distribution, loading efficiency, and cellular uptake. The optimized concentration of chitosan was 0.32 mg/mL, while the optimized concentration of hyaluronate was 0.35 mg/mL with a particle size of around 100 nm and a gene loading efficiency of around 100%, presenting appropriate properties for tumor accumulation gene therapy [37].

## 4. Chitosan Complexes for Nucleic Acid Delivery

Gene therapy can be briefly described as the delivery of exogenous genetic material into targeted cells [38,39]. To achieve this, a gene delivery vector is required. The biggest challenge in the field is the utilization of effective carriers that will not generate toxicity and immunogenicity and will promote the successful delivery of nucleic acids into human cells. These carriers can be divided into two main categories: non-viral [40,41] and viral [42] gene delivery vehicles. Both categories offer advantages and disadvantages. On the one hand, viral vectors present higher transfection efficiency; still, they exhibit increased immunogenicity issues. On the other hand, non-viral vectors are cost-effective and display reduced immune response and significantly reduced transfection efficiency [43].

**Figure 4:**
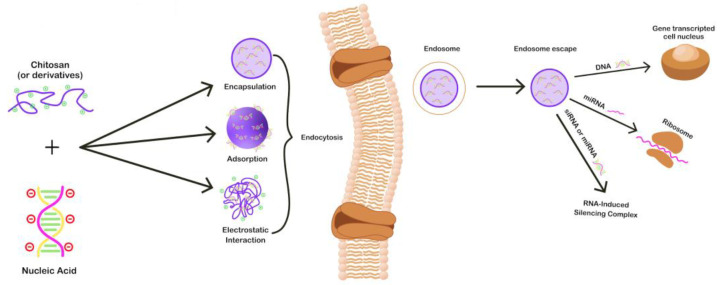
Schematic illustration of the formation of CS/nucleic acid complexes and of the transfection mechanisms of different types of nucleic acids.

In recent years, chitosan and its derivatives have gained considerable attention as non-viral carriers for the delivery of nucleic acids. Chitosan can form complexes with nucleotides through electrostatic interactions between the negatively charged primary amines present in the CS backbone and the positively charged phosphoric groups of the DNA/RNA [44]. The advantages of CS are biodegradability, increased biocompatibility, low toxicity, low immunogenicity, and a high density of positive charges that promotes the complexation with nucleotides and enhances the membrane permeability [44]. Chitosan NPs’ role as gene delivery vectors is to protect the therapeutic gene from degradation generated by endonucleases which are present in physiological fluids and simultaneously to prolong the half-life of the nucleotides, leading to increased transfection efficiency [45]. Cell uptake can occur via electrostatic interactions or passive endocytosis. Cell penetrating molecules, peptides, or ligands can be also functionalized onto chitosan NPs to further enhance this procedure [46,47,48,49]. Chitosan is also able to perform endosome escape via the so-called “proton sponge effect”. The acidic environment protonates chitosan amino groups inside the endosome, causing the drawing of water and chloride ions from the endoplasm, leading to endosome rupture [10]. As many research works have revealed, the transfection efficiency is highly influenced by a series of formulation parameters which can tune/affect the properties of CS/genetic material complexes, such as the form of chitosan used, the molecular weight, and the degree of deacetylation of chitosan as well as the pH and the N/P ratio, which can influence the size and morphology of the complexes [1,50,51]. A schematic representation of how CS NPs interact with nucleic acids as well as the transfection mechanisms are portrayed in Figure 4.

### 4.1. Chitosan–DNA Complexes

Chitosan has been used widely as a delivery vehicle for DNA. Some of the most recent works reported on chitosan/DNA nanoparticles are summarized below.

Casper et al. [52] reported on the synthesis of core-shell-structured ternary complexes for DNA delivery. Depolymerized chitosan (dCS) conjugated with linear PEI was used as the DNA encapsulating core, while dCS conjugated with PEG and cell-penetrating peptides (CPPs, sequence of 5–30 amino acids) served as the shell. The presence of PEG and CPPs induces enhanced biocompatibility and shielding of the cationic charge as well as improved DNA encapsulation and cellular uptake. The complexes were formed through electrostatic interactions between the negatively charged shells and the positively charged cores. The resulting complexes were monodispersed, with an average size of 120 nm and excellent colloidal stability. Nanovector DNA was used for the studies. The ternary complexes were characterized both in vitro and in vivo, and the results revealed improved encapsulation of nucleic acids, great transgene expression, and cellular uptake.

Dogan et al. [53] demonstrated that Artificial Neural Networks (ANNs) could be a powerful tool in our efforts to understand/predict the effect of crucial parameters (such as type of DNA, the type of the cells, concentration, *M*w) as far as the transfection efficiency of such systems is concerned in a fast, cost-effective way with fewer experiments needed. At first, PEGylated CS derivatives utilizing PEG of different molecular weights and various PEG concentrations were synthesized. Later, these systems were mixed with pDNA-encapsulated nanoparticles and crosslinker. The obtained nanoparticles were then used to modify human embryonic cells genetically, and the transfection was investigated. An ANN model was then created using the obtained experimental data, and it was proven that the above-mentioned parameters could be predicted with accuracy.

Interpolyelectrolyte complexes (IPEC) comprising DNA/CS hydrogels were prepared by Morikawa et al. [54] at various anionic-to-cationic charge ratios. CS of 50–190 kDa *M*w and a degree of acetylation of 85–95% were used along with DNA (>5 kbp). The properties of the hydrogels, both chemical and mechanical, can be tailored accordingly, as they are influenced by the anionic/cationic charge ratio. The results showed that at non-stoichiometric ratios, the DNA/CS hydrogels have the properties of the dominant component. The research group took advantage of the property possessed by both DNA and CS to bind with metals and introduced Au to the IPECs via metal-ion absorption–reduction in order to prepare a catalytic system. The catalytic activity of the Au functionalized hydrogels was proven to be increased when the CS ratio in the system was higher. The synthesized hydrogels, apart from applications in the field of biomedicine, could find potential use in other fields, such as agriculture and food science. 

PLA-PEG copolymers are one the most attractive systems for gene delivery applications, as they offer great biocompatibility. However, one major drawback is the low efficacy they demonstrate when used for such purposes. To overcome this issue, Afrouz et al. [55] introduced chitosan–folic acid (CS-FA) to PLA-PEG nanoparticles. The aim of this research was to increase the encapsulation efficiency of DNA within the nanoparticles and increase the protection of DNA from enzyme digestion damage. The latter is achieved by the co-presence of CS, FA, PLA, and PEG in the system simultaneously. MCF-7 cells were used for the purpose of this study. Increased biocompatibility, improved release of the DNA, and remarkable ability of gene transfer to the MCF-7 cells were observed.

Ma et al. [56] demonstrated that the solution pH has a significant effect on the size, morphology, charge, and compaction of DNA/CS nanoparticles. For this study, chitosan oligosaccharide lactate (average *M*_n_ = 5000) and double-stranded λ-phage DNA (48,502 b.p.) were used. The complexes were characterized with light scattering, atomic force microscopy, and magnetic tweezers. Under acidic conditions, where CS is protonated, the charge density is increased. At high CS concentrations, the electrophoretic mobility of the complexed nanoparticles remains practically the same and is dramatically reduced at elevated pHs. On the other hand, the electrophoretic mobility seems to increase at more acidic pH conditions and CS concentrations below the critical value. The obtained data were consistent when both free CS and CS/DNA complexes were measured.

Another interesting research work reports on the chemical modification of CS, aiming to improve solubility in water [57]. More specifically, CS reacted with 2-acrylamido-2-methylpropane sulphonic acid (AMP) by Michael addition. The derived CSAMP was found to be water-soluble and was able to form well-defined, small-sized particles (in the range of 150 nm) when complexed with plasmid DNA. CSAMP/DNA complexes also exhibit lower cytotoxicity and higher transfection efficiency in comparison with non-modified CS.

Bravo-Anaya et al. [27] studied the complexation of calf thymus DNA (13,000 b.p.) with CS molecules of different molecular weights (*M*w = 500,000 kDa and a degree of acetylation of 0.19 and of 0.04 and *M*w = 50,000 with a degree of acetylation of 0.04). It was found that particles formed when the higher *M*w CS was used were 280 nm in size, while smaller particles with an average size of 150 nm were formed when the lower *M*w CS was involved. Moreover, CD spectra revealed small changes regarding the conformation of the DNA, while thermal stability studies showed that the melting temperature of the DNA was elevated in the CS/DNA complexes, in comparison with free DNA. 

### 4.2. Chitosan–RNA Complexes

The delivery of oligonucleotides has been a hot topic in the last decades since it can be utilized for the treatment of several genetic diseases, cancer, or virus infections. Regulatory non-coding RNA molecules that are used in therapeutic applications can be categorized into short interfering (si), micro (mi), messenger (m), and antisense RNA. Problems arise from the solo delivery of such molecules since they are prone to degradation from nucleases in the human serum, leading to poor bioavailability and an inherent inability to enter the negatively charged cell membranes [58,59,60].

The biocompatibility of chitosan [61] and its cationic nature make it a potential candidate as a non-viral vector for RNA delivery [62,63]. Researchers have initially investigated the importance of specific parameters, such as molecular mass, degree of deacetylation (DD), and amine-to-phosphoric groups (N/P) ratio in order to improve the RNA delivery efficacy of chitosan-based polyplexes. Studies also incorporate chemical modification and peptide or polymer grafting on CS to further improve the RNA delivery abilities [11,12,64,65].

Molecular mass is a rather significant factor in the aggregation process and the formation of chitosan polyplexes in aqueous media through self-assembly. Although lower molar masses (e.g., <10 kDa) exhibit better dispersibility in aqueous media, they do not form stable nanostructures when complexed with RNA due to insufficient aggregation. In addition, low-molecular-mass chitosan creates strong electrostatic forces with RNA, preventing it from release and successful delivery [66,67]. Increasing the *M*w to over 10 kDa leads to hydrogen bonding between the –OH and –NH groups, which leads to the stacking of the chitosan molecules. Studies have shown that a *M*w of 10 kDa is the starting point of stable chitosan–RNA polyplexes in aqueous media [68]. *M*ws of ~60–140 kDa seem to have the optimal effect in terms of particle size, aggregation, complexation capacity, stability, and transfection ability, not only for plain chitosan but for chitosan/polyanions also [69,70,71,72,73,74]. The increase in *M*w subsequently leads to higher nanoparticle sizes due to extended aggregation. Nevertheless, much higher molar masses of 500 kDa have been reported in a recent work [75] utilizing galactosylated-chitosan-5-fluorouracil miRNA polyplexes for specific delivery to the liver.

The degree of deacetylation (DD) is another crucial factor for the effectiveness of the formed chitosan polyplexes that dictates the number of chitosan amino groups that are available to interact with RNA molecules. Studies have shown the importance of DD not only on the complexation efficiency and capacity of the formed nanocomplexes but also on their transfection ability [76]. A higher degree of deacetylation means more readily available amino groups that take an active part in the complexation and penetration of the cytoplasm. Studies report that a DD over 90% increased the transfection efficiency up to 80%, in contrast to 25% efficiency with DD < 80 [68].

Another important factor is the ratio of nitrogen atoms of chitosan per phosphoric atoms of RNA. The N/P ratio also dictates the free amino groups and the charged state of the whole polyplex. The higher the ratio, the more binding points are available for DNA, and better dispersibility/stabilization and transfection efficacy arise. It is very important for this ratio to be higher than 1:1, otherwise the overall charge is neutralized, all amino side groups are taken, large aggregates are formed, and precipitation is observed [68,77,78,79].

Many researchers have reported certain reactions to introduce extra hydrophilic or hydrophobic groups onto the chitosan macromolecules, depending on the application. Several ligands have been utilized, such as glutathione [80], galactose [75,81], mannose [82], aptamers [83,84], folic acid [85,86], RGD [87], and REDV peptides [88,89,90], to chemically modify the side groups of chitosan for specific cell targeting. The PEGylation of chitosan is a common strategy utilized for the increase in hydrophilicity, stability, and bioavailability, the avoidance of opsonization, and the reduction in toxicity [91,92]. Nevertheless, it was found that longer PEG chains lower the ability of the polyplexes to enter the cell and decrease the transfection efficiency [88,93,94,95,96,97,98]. Quaternization, the process of turning a tertiary amine into quaternary ammonium, has been utilized in some cases, converting the chitosan into a strong polyelectrolyte. The N,N,N-trimethyl chitosan (TMC) provides excellent stability to the formed polyplexes, since it is soluble in a wide range of pH values, while the overall transfection efficiency is improved [77,88,99,100,101]. The introduction of diethylaminoethyl (DEAE) groups is yet another chemical modification that adds more amine groups to chitosan, resulting in increased complexation capacity and transfection [102,103].

Despite all the hard work conducted in investigating the above parameters, the cell penetration and cell uptake abilities of the chitosan/RNA polyplexes are yet to be improved [104]. Hybrid/chimeric nanostructures have been synthesized by grafting peptides or synthetic polymers onto chitosan. Several important studies have been conducted in this field, utilizing synthetic or natural cell-penetrating peptides, such as histidine [46], TAT, TAT-trans, CGKRK, arginine [46], nona-arginine [47], poly-l-arginine [48], and protamine [49]. The conjugation of such peptides might need the use of a linker molecule, such as glycine [105], glycol [106], PEG [94], or fatty acids [107,108]. The incorporation of such peptides led to increased complexation and better protection of RNA from serum protease. The most important result was the much higher cellular uptake of such polyplexes, while the transfection and gene silencing ability were significantly improved.

The use of other polysaccharides, such as dextran [69] and hyaluronic acid (HA) [70,109,110], has been also reported, either as simple complexation with chitosan or via ionic gelation in order to improve biocompatibility, solubility, and stability. This process refers to the ionic complexation with the utilization of a crosslinker molecule such as tripolyphosphate (TPP), and it was reported to offer better stability and stronger interactions between chitosan and RNA [98,111,112]. Another study reports the complexation of RNA with a triple polysaccharide system of chitosan/hyaluronic acid and chondroitin sulfate, crosslinked via ionic gelation [113]. 

Conjugation with polyethyleneimine is also another idea for improving chitosan’s properties for gene delivery. PEI is a synthetic polymer comprised of an amine group and two carbon aliphatic CH_2_CH_2_ spacers in its main chain and is widely known as a non-viral gene delivery agent since it bears strong cationic charges. Despite its impressive abilities for such applications, it exhibits a rather high toxicity on its own. Conjugation with chitosan though leads to the best of two worlds, with significantly decreased toxicity and enhanced gene delivery abilities [114,115,116].

## 5. Applications of Multifunctional Chitosan-Based Nanoparticles in Pharmaceutics, Medicine, and Precision Medicine

As mentioned previously, chitosan is a safe biomaterial and pharmaceutical multifunctional excipient with several applications in gene delivery. Several routes of administration have been already reported for chitosan-based nanoparticles, including oral, intravenous, intramucosal, nasal, and transdermal delivery, as well as targeting to brain [22,23,24,25,26,117]. Furthermore, chitosan–nucleic acid complexes are widely used for cancer therapy, photothermal cancer therapy, vaccine platforms, and adjuvants [22,23,24,25,26]. Here, we are going to discuss some of the multifunctional chitosan-based nanoparticles and their applications in the fields of pharmaceutics, medicine, and precision medicine. A summary of the most recent studies with applications and outcomes is also presented in Table 1 at the end of this section.

Chitosan Au nanorods were loaded with siRNA for the treatment of triple-negative breast cancer. Tail vein injection in animals showed improved effectiveness through photothermal ablation (significant synergistic effect) [118]. *N*-succinyl chitosan-poly-l-lysine-palmitic acid micelles stable in conditions of human plasma were loaded with doxorubicin and P-glycoprotein siRNA for reversal of multidrug resistance and synergistic effect in human liver cancer in cell lines and in animal studies [119]. Nanoparticles composed of low-molecular-weight chitosan were co-loaded with methylprednisolone and plasmid DNA for the reduction in inflammation at the injury site and future treatment of spinal cord injury [120]. Xiao et al. [22] prepared a chitosan-based hydrogel for the co-delivery of TNFα gene silencing siRNA and interleukin-22 for the therapy of ulcerative colitis via oral administration. Chitosan-coated polyplexes for the co-encapsulation of multidrug-resistance-inhibiting siRNA and doxorubicin were used for the improvement of the therapy of multidrug-resistant tumors [121].

Additionally, it has been reported that chitosan enhances the gene delivery of oligonucleotide complexes with magnetic nanoparticles and incorporated cell-penetrating peptides [122]. Namely, cell-penetrating-peptide-conjugated chitosan-modified iron oxide magnetic nanoparticles were loaded with genetic material with increased colloidal stability and transfection efficiency. Chitosan nanoparticles were investigated for vaccination against viral infections as sub-unit vaccines and as adjuvants platforms too [1]. The mechanism relies on the transfer of small oligonucleotides to the virus core, changing its mRNA sequence, hence disabling vital abilities, such as cell binding, replication, etc. [79]. In addition, the transfer of oligonucleotides to macrophages is another way to enable an immune response for certain viruses and bacteria [34,123]. Chitosan derivative nanoparticles enhanced the immunogenicity of a DNA vaccine encoding hepatitis B virus core antigen in mice [124].

Methylenetetrahydrofolate dehydrogenase 1-like (MTHFD1L) shRNA was encapsulated into chitosan/tripolyphosphate nanoparticles with a size of around 150 nm with efficacy on the gene expression of oral squamous cell carcinoma cells in combination with photodynamic/gene therapy [125]. Fernández-Paz et al. [126] proposed chitosan-based nanocapsules for pulmonary gene delivery to promote gene-transfection in the lung epithelium. The plasmid encapsulation efficiency was more than 90%.

As mentioned above, there are several examples of chitosan-based nanoparticles for nucleic acid delivery in different diseases and vaccines. The biocompatibility and low immunogenicity of chitosan in comparison to other cationic polymers which exhibit a toxicity profile make it a pharmaceutical excipient with added value for clinical translation. The formulation versatility, the high loading capacity of nucleic acids, the co-delivery of drugs and genes, the physicochemical characteristics, and the colloidal stability are some of the advantages of chitosan-based delivery carriers. Additionally, the mechanism of internalization by creating tight junctions between the epithelial cells is also another advantage of this biopolymer due to its penetration-enhancing mechanism [22,23,24,25,26]. Furthermore, drug delivery nanosystems and those composed of chitosan have inherent toxicity, primarily as a result of their nanosize and nanoparticulate nature but also occasionally as a result of their ADME(T) and pharmacokinetic profile. Although most of the systems discussed in this manuscript are thought to be biocompatible, it is crucial to undertake in vitro and in vivo nanotoxicity and immunogenicity studies in order to verify the biosafety of the systems [127]. Namely, according to the recent literature, any biopolymer’s viability for drug delivery purposes is largely determined by how it will be metabolized in the human body [128]. Generally, chitosan is considered a safe material, i.e., chitosan’s cytotoxicity toward human lymphoblastic leukemia and human embryonic lung cells was negligible when tested in vitro [128]. There is a report in the literature where an intravenous chitosan dose of 50 mg/kg was found to be lethal, perhaps because of blood aggregation [129]. On the other hand, there are some limitations and challenges associated with chitosan-based nanoparticles. The stability, scalability, immunogenicity, and potential nanotoxicity are the main problems that should be solved in their preclinical evaluation. The therapeutic stability of both nucleic acids and nanoparticles is also a crucial issue for the design of nanomedicines. The physicochemical degradation pathways, the stability of the dispersion state, and the extracellular and intracellular delivery issues are of paramount importance for the development and high-end manufacturing of nanopharmaceuticals [130,131]. Regarding scalability, large-scale production is costly, and several physicochemical and morphological techniques, as well as methods to prove loading and encapsulation efficiency (%), should be addressed to monitor and prevent batch-to-batch variability. Even though there are many chitosan-based nanoparticles as gene carriers in the literature with significant results in the fields of nanomedicine, there have not been yet any formulation in clinical trials. The formulations that are currently on the market or in clinical trials belong to the category of medical devices. Some authors believe that there are some limitations for the clinical translation of chitosan, such as physiological pH deduction and poor targeting capacity.

Taking all of the above into account, chitosan-based nanoparticles exhibit great potential and impact in the larger context of biomedical research due to several reasons. Firstly, there is a deeper understanding of the interactions between chitosan and nucleic acids during the pre-formulation studies. Secondly, the design and development of biopolymer-based nanoparticles are very useful in the fields of pharmaceutical technology, materials science, and nanotechnology. Thirdly, several physicochemical, morphological, and thermodynamic techniques are used for the full characterization of these multifunctional nanostructures, according to the requirements of the regulatory framework. Last but not least, new techniques and processes are created in order to prevent and monitor batch-to-batch variability, leading to high-end manufacturing and the adoption of the “Quality-by-Design” overview in the pharmaceutical industry [132]. Chitosan and, in general, the biopolymers meet the criteria for materials that can be used as excipients with several properties for the development of advanced drug delivery platforms with added value for patients.

**Table 1:** List of recent studies regarding chitosan–oligonucleotide polyplexes, applications, outcomes, limitations, and future perspectives.

Polyplexes	Aim of the Study	Application	Study Outcome	Limitations	Future Research
CS-dsRNA [63]	RNA interference (RNAi) in fall armyworm (FAW), Spodoptera frugiperda	Pesticides	Chitosan helps endosome escape and protects dsRNA	Not as effective as cellfectin II transfection reagent	Better formulation, better efficiency, cellular uptake, and biodistribution
CS-dsRNA [133]	RNA interference (RNAi) in Caenorhabditis elegans	Pesticides	Chitosan NPs under real environmental conditions	Not useful in elevated pH and natural organic material conditions	Durable materials against high pH values and real environmental conditions
CS-siRNA [62]	Silencing of the lncRNA NEAT1 expression vector	Colon cancer	High transfection to colon cancer cells, growth inhibition and accelerated apoptosis	-	In vivo research
CS-microRNA [78]	Downregulation of MCF-7 cell mRNA expression	Breast cancer	*M*_w_ ~40 kDa, DA ~12%, N/P ratio = 1.5 for particles, N/P = 8, and DA of 30% for transfection	-	Conditions must be further optimized, cell penetration mechanism to be studied further. In vivo research
CS-Zn-miR-224 [86]	Delivery of LNA-miR-224 to colon cancer cells	Colon cancer	-	-	-
CS-CMD-miR-145 [69]	Delivery of miR-145 to breast cancer cells	Breast cancer	Many parameters were tested for stability, biocompatibility, and transfection	High CMD-Chi ratio, better stability; lower ratio, better transfection	In vivo studies, better optimization with the known parameters
TMC-g-PEG-VAPG/miRNA-145 [101]	Transfer of miRNA to SMCs	-	Low cytotoxicity, RNA condensation, great transfection to SMCs, controlled proliferation after 56 days of release	-	In vivo studies should be performed
tCS/nHAp/nZrO_2_-miR-590-5p [134]	Transfer RNA to MSCc for bone regeneration	Bone regeneration	Activating different signaling pathways that promote osteogenesis		In vivo studies should be performed
CS-Glu-TA-miRNA-219a-5P [80]	Brain delivery of miRNA219a-5P	Multiple sclerosis	miR-219 overexpression, crystallin alpha B upregulation, apolipoprotein E downregulation, lower inflammation	Lack of dual-luciferase reporter and Western blot assays for better understanding of the underlying mechanisms	Further clinical trials involving different species should be performed
CS-microRNA-222-Silf fibroin scaffolds [135]	Transfer miR-222 to NSCs for neural tissue regeneration	Neural tissue regeneration	High RNA encapsulation efficiency, enhancement of NSCs proliferation	-	In vivo studies to be performed
Chi-Echinococus miRNA [123]	Echinococus-miRNA delivery for antibacterial treatment	Antiviral vaccines	Protection from miRNA degradation, stability, low cytotoxicity, efficient transfection, reduction of UBE2N in the liver, potential target of emu-miR-4989	-	Further clinical trials to be performed
CMD-Tocopherol-miRNA-218 [136]	Transfer of miRNA-218 to GIST cells	Gastrointestinal stromal tumor	Spherical size ~110 nm, inhibit cell proliferation, superior cell apoptosis	-	In vivo studies to be performed
CS-Chi-HA-microRNA-149-5p [113]	Transfer of microRNA-149-5p for cartilage regeneration	Osteoarthritis	Non-toxic, increased microRNA-149-5p and decreased FUT-1 levels, efficient transfection, enhanced chondrogenesis	-	In vivo studies to be performed
CS-g-PGM-Dex-PEI-LTX-315-melitin-miR-34a [137]	Transfer of microRNA and cytotoxic peptides to breast cancer cells	Breast cancer	Spherical size 123 nm, no cytotoxicity, smart targeting, good encapsulation efficiency, synergistic effect of increased cancer cell death	-	In vivo studies to be performed
CS-microRNA34a [138]	Transfer of microRNA34a to breast cancer cells	Breast cancer	Spherical NPs of 135 nm, target cell uptake, no cytotoxicity, miR-34a upregulation, inhibit growth, migration, and invasion of cancer cells	-	In vivo studies to be performed
CS-TPP-miR-33 [98]	Transfer miR-33 to macrophages to lower LDL cholesterol	Cardiovascular diseases	Biocompatible, efficient transfer to macrophages, regulate ABCA1 expression and cholesterol efflux	-	Same NPs could be used for atherosclerosis treatment
CS-miR [139]	Transfer two types of microRNA to SKOV3 ovarian cell line	Ovarian cancer	Good biocompatibility, transfer of both microRNAs to target, suppression of GLI1	-	In vivo studies should be performed
CS-microRNA [140]	Transfer microRNA-219 to human GBM cell line (U87 MG)	Gliobstatoma	Biocompatibility, high entrapment efficiency, increased reduction of growth after 48 h	-	In vivo studies to be performed
CS-microRNA [141]	Transfer of miR-144/451a	Oral cancer	Enhanced protection of RNA, reduced viability, migration, and invasion of cancer cells	-	In vivo studies to be performed
CS-TiO-miRNA [142]	Transfer of antimir-138 to MSCs for bone regeneration	Osteogenesis	Sustained release over 2 weeks, efficient cell uptake, even distribution on the surface, good biocompatibility, increased MSCs differentiation and osseointegration	-	Additional clinical studies to be performed
CS-DTX-anti-microRNA [97]	Transfer of anti-miR-21 to breast cancer cells	Triple-negative breast cancer	Spherical NPs of 90 nm size, high entrapment efficiency, good transfection ability, stability, and protection of RNA, blocking of miR-21 expression	-	In vivo studies to be performed
CS-miR-141 [143]	Transfer of miR-141 to breast cancer cells	Breast cancer	Metastasis, VEGF, EMT, and invasion were significantly reduced, increased apoptosis up to 2.5 times	-	In vivo studies to be performed
CS-siRNA/hsDNA [79]	Antiviral activity inhibiting HIV-1 proliferation	HIV-1	Stable complexes, good transfection efficiency to infected cells, high viral inhibition	HIV-1 mutations	Mixture of different siRNAs to target more HIV-1 gene sequences
CS-pDNA [144]	Transfer of pDNA for cartilage regeneration	Osteoarthritis	Increased chondrosynthesis, decreased nitric oxide, ADAMTS-5, and MMP-13 levels	-	Further studies in different cell species are needed
CS-p53 targeting pDNA [145]	p53 protein expression on HeLa cells	Cancer HeLa Cells	Biocompatibility, high complexation and transfection efficiency, good p53 upregulation	Upregulation of p53 can be even higher	In vivo research is needed
Chi-pDNA [34]	pDNA delivery to macrophages as anti-rabies vaccine	Anti-rabies vaccine	120 nm size of NPs, 100% attachment efficiency, biocompatible, improved pDNA transfection	-	More clinical trials have to be performed
Chi-AMP-DNA [57]	DNA delivery to A549, HeLa, and HepG_2_ cancer cells	Cancer	Enhanced DNA encapsulation, high transfection efficiency	-	Additional clinical trials to be performed
Chi-PLA-PEG-FA-DNA [55]	DNA delivery to MCF-7 cancer cells	Cancer	Biocompatible, good transfection, high encapsulation efficiency	-	In vivo studies to be performed

## 6. Conclusions

The biocompatibility and properties of chitosan, as well as its versatility, make it ideal for applications in medicine, pharmaceutics, and precision nanomedicine. In this review, we discussed the physicochemical properties of chitosan and its biocompatibility and safety, which make it an ideal material for medicinal applications. The techniques, protocols, and properties, as well as all the technological considerations of chitosan complexes for nucleic acid delivery, were also presented. Special attention was given to the formulation parameters for the design and the development of chitosan-based polyelectrolyte nanoparticles for gene delivery. Examples from the recent literature on chitosan–DNA and chitosan–RNA complexes were discussed in terms of their characteristics and added value for targeting tissues and cells. Considering the recent preclinical evaluation of chitosan-based nanoparticles for nucleic acid delivery and targeting, the fast clinical translation of these polysaccharide-based platforms is coming in the next years.

## Data Availability

Not applicable.
